# tagtog: interactive and text-mining-assisted annotation of gene mentions in PLOS full-text articles

**DOI:** 10.1093/database/bau033

**Published:** 2014-04-07

**Authors:** Juan Miguel Cejuela, Peter McQuilton, Laura Ponting, Steven J. Marygold, Raymund Stefancsik, Gillian H. Millburn, Burkhard Rost, the FlyBase Consortium

**Affiliations:** ^1^Goerresstr. 20, Munich 80798, Germany, ^2^Department of Genetics, University of Cambridge, Cambridge CB2 3EH, UK, ^3^Department of Informatics, Technical University of Munich (TUM), Garching 85748, Germany

## Abstract

The breadth and depth of biomedical literature are increasing year upon year. To keep abreast of these increases, FlyBase, a database for Drosophila genomic and genetic information, is constantly exploring new ways to mine the published literature to increase the efficiency and accuracy of manual curation and to automate some aspects, such as triaging and entity extraction. Toward this end, we present the ‘tagtog’ system, a web-based annotation framework that can be used to mark up biological entities (such as genes) and concepts (such as Gene Ontology terms) in full-text articles. tagtog leverages manual user annotation in combination with automatic machine-learned annotation to provide accurate identification of gene symbols and gene names. As part of the BioCreative IV Interactive Annotation Task, FlyBase has used tagtog to identify and extract mentions of *Drosophila melanogaster* gene symbols and names in full-text biomedical articles from the PLOS stable of journals. We show here the results of three experiments with different sized corpora and assess gene recognition performance and curation speed. We conclude that tagtog-named entity recognition improves with a larger corpus and that tagtog-assisted curation is quicker than manual curation.

**Database URL:** www.tagtog.net, www.flybase.org

## Introduction

A major challenge facing biological databases today is the increase in data available for curation. Concurrent with an increase in the number of biological journals, there has been a movement from printed literature to web-based HTML and PDF. This has removed many of the financial and technical constraints on the length and the number of articles a journal can publish. For the past 30 years, the number of Drosophila-related primary research articles published each year has steadily increased from ∼1000 in 1980 to >2000 a year since 2001 (1). FlyBase (http://flybase.org) is the premier database of *Drosophila melanogaster* genes and genomes (2) and manually curates Drosophila-related information from the published literature. This information hangs from genetic objects, such as genes, alleles and transgenic constructs. Our genetic literature curation pipeline has two main stages: (i) skim or author curation, where the genes in a paper are identified, and flags are added to indicate the presence of a new reagent or data type (e.g. a new allele or gene expression in a perturbed background), and (ii) full curation, where all other genetic objects are added and annotated with phenotypic, molecular, expression and interaction data. Manually curating each gene mentioned in a paper is a time-consuming process and takes a significant amount of curator effort. Finding a way to automate this process would greatly increase curation efficiency, not to mention the number of papers that could be fully curated.

**Figure 1.**
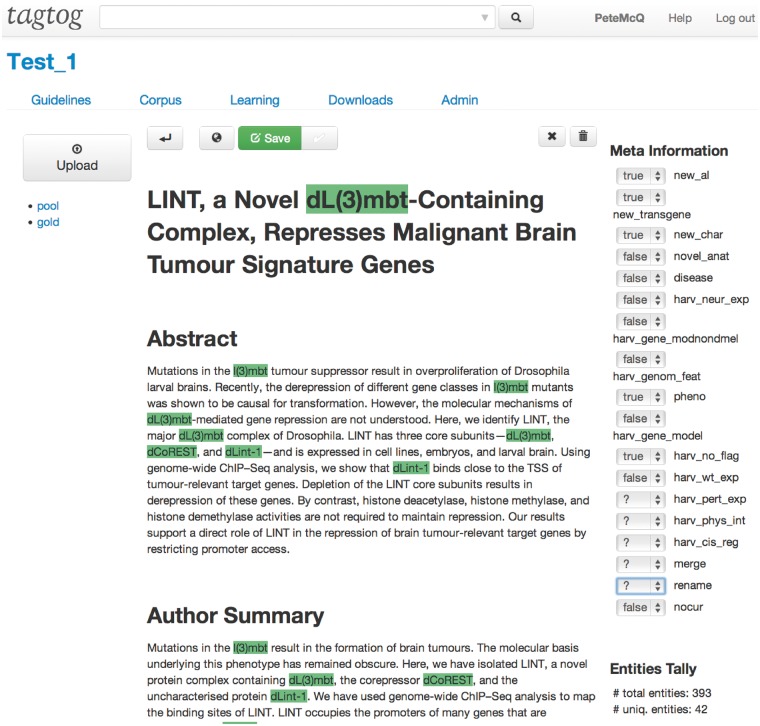
Example of the document display and editor in tagtog.

Since the meeting at the BioCreative workshop in 2012, FlyBase has been collaborating with tagtog to identify and extract Drosophila gene mentions from PLOS journals (3). tagtog (http://tagtog.net) is a web-based framework for the annotation of named entities. The tagtog system allows biocurators to annotate gene symbols manually and leverages machine learning methods to perform the same type of annotations computationally (Figure 1). Initially, the tool is trained with a small set of manually annotated documents. tagtog can then be used to process a set of novel documents wherein automatically generated predictions are made, which can be reviewed and validated by the user. This continuous and interactive retraining of the machine learning methods with user feedback can lead to an ever-improving performance in automatic prediction (4). Once optimized, the trained machine learning methods can be used to process and annotate a large volume of documents to a sufficiently accurate level.

In this collaboration between FlyBase and tagtog, we have annotated >450 PLOS journal articles and explored whether the size of the annotated corpus affects the precision and recall of automatic named entity recognition (NER) and whether NER can speed up gene symbol and name annotation.

### The tagtog system

In the following section, we briefly showcase some of tagtog’s main features.

- **Multiple projects:** users can create different annotation projects and load their own dictionaries and corpora.
- **Team collaboration:** multiple users on the same project are also supported, allowing curation teams to view and annotate the same set of documents.
- **Entity normalization:** entities (such as gene names) can be normalized to unique identifiers (IDs) using a reference dictionary submitted by the user.
- **Active learning:** tagtog actively asks for user feedback on predicted annotations. A proposed mechanism was already developed in an early version of tagtog, presented at the BioCreative 2012 workshop (5).
- **Document searching:** papers can be searched using the search tool at the top of the interface. Options include searching by document ID (based on the digital object identifier), entities or whether a paper has been fully annotated. In the future, we hope to add the facility to search by PubMed ID (PMID).
- **Browser support:** the system runs on all major current browsers only requiring HTML5 and javascript. Chrome and Firefox are officially supported. Other browsers like Opera, Safari and Internet Explorer (9 and 10) are regularly tested but lack official support at this point.
- **Import options:** any paper following the NCBI Journal Publishing Tag Set (6) or the BioMed Central format (7) can be uploaded to tagtog. This includes full-text papers from the PLOS, BioMed Central, Chemistry Central and Springer Open collections. In the near future, we will accept papers from the new JATS format (8) and plain text files.
- **Export options:** three export file formats are supported: a tab-separated list of terms linked to PMIDs (TSV format), the new BioC format (9) and ‘anndoc’ XML, our in-house format. Further file formats can be added on request.

### Defining the annotation guidelines

**Figure 2.**
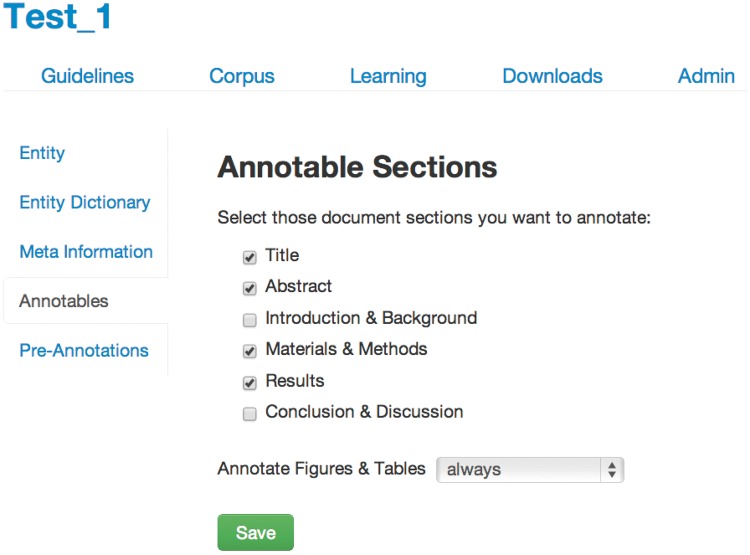
Annotation guidelines.

On project creation in tagtog, the first step for a user is to define the annotation guidelines (Figure 2). These guidelines stipulate what should be annotated and how this relates to the entity class. There are the following options:

- **Entity:** choose the entity class name to annotate. For this project, we chose to annotate all *D. melanogaster* gene mentions, both as symbols (for example, ‘dpp’ or ‘amn’) and names (for example, ‘decapentaplegic’ or ‘amnesiac’), where the gene is a separate string or is separated from another entity by a hyphen. We also included some non-Drosophila genes, such as the commonly used GAL4 drivers from the UAS-GAL4 system (10) and expression markers such as GFP, RFP and lacZ.
- **Entity Dictionary:** upload a user-defined dictionary of collected entity names. The dictionary can contain synonyms and database-specific IDs, allowing data integrity checks and seamless integration of the results with the parent database. We generated a dictionary of FlyBase gene symbols, gene names and gene symbol and name synonyms based on the ‘FB_2013_05 release fb_synonym_fb_2013_05.tsv.gz’ file available from the files download page on the FlyBase Web site (11).
- **Meta Information:** define a list of checkboxes for document triage, e.g. whether the article contains human disease mentions or information on a new transgene. We generated checkboxes for all the FlyBase triage flags, so the annotation of the tagtog corpus could be used directly in the FlyBase curation.
- **Annotatable material:** select the sections of the full-text articles that can be annotated and trained on. The annotation of captions from figures and images can be decided independently: ‘always’, ‘never’ or ‘section-dependent’*.* For this project, we annotated the title, abstract, materials and methods, results and figure legends. We did not annotate gene mentions in the introduction or the conclusion/discussion sections, as per FlyBase curation rules.
- **Pre-Annotations:** users can activate or deactivate this feature. Pre-annotations are annotations that are automatically generated within an individual document when a user adds or removes an annotation (i.e. selects or deselects a word). These automatic annotations are generated as follows: if a user selects the entity ‘X’, in the same document all mentions of ‘X’ will be pre-annotated and assigned to the same entity class. The converse is true for deselections. Note that the automatic pre-annotations are not machine learning-based but simple matches of equal strings. The pre-annotations are marked with a special flag and have to be validated or removed by the user before the containing article can be used for training.

### The machine learning component of tagtog

A core defining characteristic of the tagtog system is that the users can choose the entity class to annotate, such as genes, Gene Ontology terms or diseases. The system boasts a general-purpose named entity recognizer implemented with conditional random fields (CRFs) (12). For the biomedical domain, the CRFs are trained with common features used in previous systems. However, in contrast to best performing methods like AIIAGMT (13), which use the aggregation of various CRF models, we use one sole backward model. This results in a slightly lower performance but has the benefit of an increased speed, which is essential in a user-interactive application. The recognizer can be customized to the prediction task at hand by means of user feedback and by using a dictionary of entity terms. The system can also be expanded with new machine annotators via plug-ins to enable annotation of diverse classes and domain languages within the same document. If desired, the machine learning component of tagtog can be turned off to allow biocurators to use the tagtog interface exclusively for manual curation.

### Defining the project corpus

Every project in tagtog manages a corpus of documents, which can be uploaded either individually or in batches. The system's internal parser recognizes the documents' sections, subsections, figures, tables and some additional meta-information such as the paper's original uniform resource locator (URL). The project corpus can be augmented progressively as the user sees fit. Currently, documents are placed in two folders, the ‘pool’ folder, where most documents are placed, and the ‘gold’ folder, where a smaller set of manually annotated documents is used exclusively for the evaluation of the machine learning methods’ performance. Only the documents in the pool folder can be used for training.

### Generating the FlyBase corpus

To date, FlyBase curators have manually annotated 451 full-text articles using the tagtog interface. The PLOS journal collection was chosen for document sampling because PLOS makes all their research papers fully available for text mining (14), and the PLOS journal collection covers many aspects of Drosophila research. All sampled papers are from between 2011 and 2013. The following document sections were annotated: title, abstract, results, materials and methods and figure and table legends. The paper annotations have been used to iteratively train the machine learning component of tagtog. So far, we have performed three annotation and benchmark iterations. In the first two iterations, annotations were done manually by a sole curator and automatically by the system. In the third iteration, all five FlyBase curators annotated papers manually. All the manual annotations and corrections were performed using tagtog's document editor interface.

**Iteration 1**: a sole curator (P. McQuilton) manually annotated a training set of 20 articles, representative of the Drosophila-related papers found in PLOS journals. The number of 20 ‘seed’ articles was chosen based on best practices by previous experiments on active learning (15). We searched the PLOS Web site using the term ‘*Drosophila melanogaster*’ from 2011 onward and then randomly selected 20 articles that had been already annotated and incorporated into the FlyBase database. Trained with these documents, the system was applied to predict gene mentions in an unlabeled validation set of 99 articles. The curator then went through the validation set and corrected, added or removed the predicted annotations, when appropriate. Mismatched annotations between the original predictions and the revised annotations were counted as errors.

**Iteration 2**: the two sets of papers used in Iteration 1 were united to form a training set of 119 articles. For evaluation, the user manually annotated a test set of 20 new articles (which we will refer to as the ‘Gold Standard’. The system was retrained on the 119 articles and benchmarked against the 20 Gold Standard articles. In contrast to Iteration 1, prediction errors could be compared directly against the test set.

**Iteration 3**: the previous two sets, plus a further 312 papers curated by five different FlyBase curators, were combined to form an annotated corpus of 451 fly-related papers. These papers were used to retrain tagtog before the assessment on the Gold Standard set (20 papers).

### Measuring performance on the FlyBase corpus

We used standard NER evaluation measures to benchmark performance, namely, precision (P), recall (R) and F1 measure (F1). Precision measures the percentage of correct predictions, i.e. the number of correct predictions divided by all predictions. Recall measures the percentage of correctly identified entities, i.e. the number of correctly identified entities divided by all entities present in the test document. There is typically a trade-off between precision and recall; F1 averages the two into one sole measure. More precisely, F1 is the harmonic mean between precision and recall. Only exact matches between the ‘tagtog’ predictions and the test annotations are counted as correct, i.e. the predictions have to match the exact word boundaries [for example, ‘Su(H)’ but not ‘Su(H) protein’]. Two types of counts were considered: (i) unique entities on a document basis. That is, for a test entity X in a document, the predictions are right if at least one mention of that entity can be identified in that document, wrong otherwise (for example, at least one mention of the gene ‘dpp’ is correctly identified, no matter whether other mentions may be missed). Equivalently, all unique entities identified by the predictions but not present on the test annotations are counted as errors. (ii) All entity mentions for all documents. That is, for all entity mentions, matching predictions and test annotations are counted as correct, whereas mismatched mentions, either false-positive findings or false-negative findings, are counted as errors (so in this case, three correct mentions of ‘dpp’ can be identified, while one mention is missed and recorded as a false negative). Note that for testing, only the annotatable sections defined by the curator are compared.

**Figure 3.**
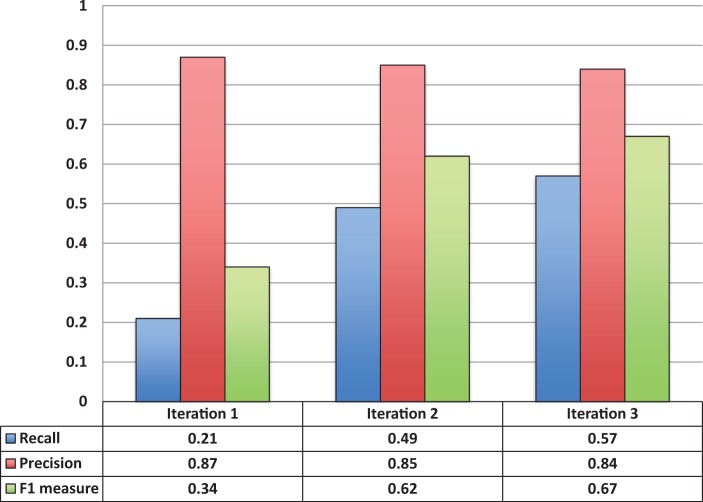
Entity recognition performance over all three corpora sizes.

**Figure 4.**
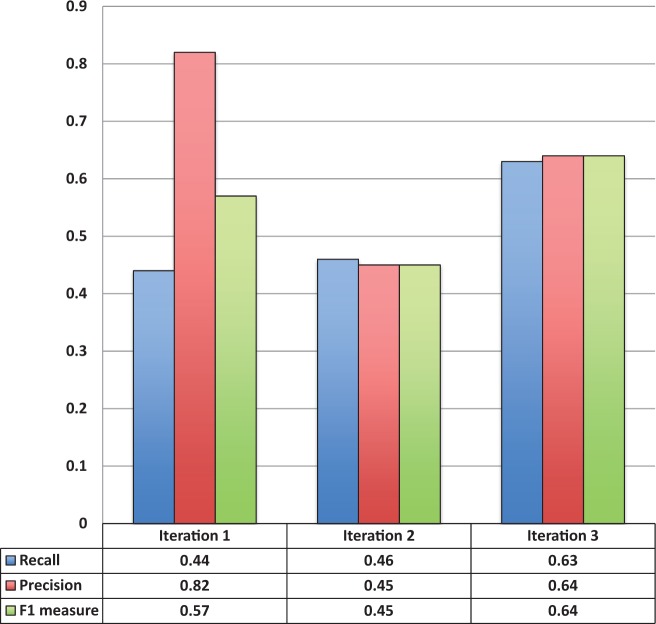
Unique entity recognition performance over all three corpora sizes.

Figure 3 shows the entity recognition performance for all entity mentions in a paper, i.e the ability of tagtog to identify the presence of a gene mention, either as a symbol or name. The figure shows that the performance has steadily improved (taking the F1 measure) in proportion to the corpus size. The same performance improvement behavior is seen for unique entity recognition (Figure 4), that is, the ability to identify the presence of a gene at least once in a paper. In this case, however, we found a large reduction in precision performance from Iteration 1 (P = 0.82) to Iteration 2 (P = 0.45). We observed numerous false-negative findings that were repeated only once in the text, examples: ‘BamH1’ in ‘journal.pgen.1003042’ or ‘oskar’ in ‘journal.pgen.1003079’. False-negative findings can significantly impact performance of unique entities, but leave the performance of all mentions mostly unaffected if the unique false-negative findings represent a small fraction of the total number of mentions. Nevertheless, in Iteration 3, both the precision and the recall for unique entities increased considerably (P = 0.64 and R = 0.63).

The final number of 451 papers consists of a test set of 20 manually annotated documents plus a training set of 431 documents, which combine manual and automatic annotations (that have subsequently been manually validated). We have deposited this corpus in the BioC format (7) at the BioC repository (http://www.ncbi.nlm.nih.gov/CBBresearch/Dogan/BioC/) for use by other text-mining groups. We believe the corpus to be the largest and the most complete gene mention annotation set in full-text articles currently available.

### The BioCreative interactive annotation task challenge—curation time

Within the framework of the BioCreative IV workshop, the purpose of the interactive annotation task (IAT) was to ascertain the possible benefit in terms of curation effort of machine-assisted annotation versus manual annotation. The task for tagtog was divided as follows:

1. Manual annotation: using the tagtog interface, a biocurator manually annotated a set of 20 documents with an entity class of her choice. The machine learning component of tagtog was consequently trained on the first manual set and provided automatic annotations for a second set of 20 documents.
2. Assisted annotation: using the tagtog interface, the biocurator reviewed and corrected, where appropriate, the automatic predictions of the second set of 20 documents.

Curation time was measured for both subtasks, and the results were compared. Two biocurators participated in the task, Mary Schaeffer from MaizeDB (first) and Ritu Khare from NCBI (second):

- The first biocurator chose to annotate maize-related genes and uploaded a self-defined dictionary of terms. She is an expert in this kind of annotation. A total of 6 h and 34 min was taken for the manual annotation and 4 h and 5 min for the assisted annotation. This indicates a reduction in curation time of ∼1.6-fold.
- The second biocurator chose to annotate Drosophila gene names and symbols and uploaded the same dictionary as used with the FlyBase corpus. The second curator is not an expert in this kind of annotation. She spent 9 h and 19 min for the manual annotation and 4 h and 49 min for the assisted annotation. This indicates a reduction in curation time of ∼1.9-fold.

## Conclusions

We have shown that tagtog can be used successfully to annotate Drosophila gene symbols and names. We have also shown that the accuracy of these annotations increases with the size of the training corpus. In addition, we have shown that tagtog-assisted NER can reduce overall curation time. This gradual improvement in accuracy, combined with the shortening of curation time by 1.6- to 1.9-fold compared with completely manual curation, illustrates the benefit of including text-mining techniques, such as tagtog, in curation. To our knowledge, these preliminary results represent one of the first NER evaluations with a substantial amount of full-text articles in the biomedical field.

Given the encouraging nature of the curation time experiments, we plan to expand our analysis of curation with tagtog to assess whether the increase in curator speed is due to familiarity with the tool or assisted annotation. These experiments have also shown that tagtog can be used to annotate gene symbols from species outside of Drosophila, such as maize.

In future work, we will check for the presence of repeated entities between documents that could bias the NER evaluation between iterations and assess inter-annotator agreement between the five FlyBase curators to allow performance benchmarking. NER with full-text articles is understood to be considerably more difficult than for abstracts (16, 17), and although we have not specialized the machine learning methods used here for Drosophila gene mention extraction, we are pleased with the level of performance. The continuous learning of tagtog is designed to generate cheaper (in terms of manual curation effort) training data, by taking advantage of semiautomatic annotation. We will continue to add to the FlyBase corpus, with the aim of increasing NER accuracy and the potential incorporation of tagtog (or the output from tagtog) into our genetic literature curation pipeline.

In this article, we have illustrated how tagtog-assisted annotation can benefit manual curation from the literature. We have shown how the identification of *D. melanogaster* gene symbol and name mentions has gradually improved with more training data and user feedback. This illustrates the adaptability of the tagtog system to the specific curation requirements of the user, and there seems to be a potential for further improvement in NER performance. Thanks to our participation in the BioCreative IV IAT challenge, we have been able to achieve promising results in the reduction of curation time through the use of tagtog-assisted curation compared with manual gene mention extraction. As a result of our experiments, we have generated the FlyBase corpus, one of the largest corpora of full-text articles with entity annotations in the field of biomedical text mining. We have made this available in BioC format for use by the text-mining community.

## Author contributions

J.M.C. and P.M. devised the experiments and wrote the article. P.M., S.M., L.P., R.S. and G.M. annotated the corpus and provided feedback on the tagtog interface. J.M.C. developed tagtog.
